# Supplementary material for: Biodiversity and Ecosystem Function under Simulated Gradient Warming and Grazing
Source: Plants (Basel). 2022 May 27;11(11):1428. doi: 10.3390/plants11111428 (PMC9182780; doi:10.3390/plants11111428)
Supplement: Supplementary file 1 [file plants-11-01428-s001.zip › Highlights.pdf]

(1) Plant diversity varied non-linearly with progressively increasing warming, with the main influence on change being soil moisture content.
(2) Soil bacterial diversity was significantly affected by warming and soil fungal diversity was significantly affected by grazing.
(3) Temperature rise caused biomass transfer to the subsurface, moderate temperature rise (approximately 1°C) increased biomass, light grazing did not reduce biomass, and community stability was not affected by temperature rise or grazing.
(4) Temperature influence biomass more strongly than biodiversity. Community stability was not significantly correlated with biomass.
