# Supplementary material for: Biodiversity and Ecosystem Function under Simulated Gradient Warming and Grazing
Source: Plants (Basel). 2022 May 27;11(11):1428. doi: 10.3390/plants11111428 (PMC9182780; doi:10.3390/plants11111428)
Supplement: Supplementary file 1 [file plants-11-01428-s001.zip › Supplementary Materials.pdf]

Table S1 Effects of gradient warming and simulated grazing on environmental factors, biodiversity and ecosystem function

	Gradient warming					Grazing		W×G
	CK	A	B	C	D	NG	G	
T.AG5	1.14±0.87b	1.09±0.79b	3.18±1.62a	2.09±1.13ab	2.16±0.59ab	1.72±0.95a	2.15±1.53a	*P*=0.24
T.AG10	1.55±1.91a	1.48±2.15a	2.48±1.69a	2.87±1.61a	3.38±2.19a	2.35±2.15a	2.36±1.87a	*P*=0.59
T.BG5	3.56±2.74a	3.48±2.15a	4.15±2.31a	4.23±2.62a	3.66±2.42a	4.16±2.42a	3.47±2.33a	*P*=0.69
SMC	38.53±3.45a	37.28±2.38a	36.78±2.82a	37.55±2.35a	33.81±2.59b	37.34±3.28a	36.24±2.84a	*P*=0.96
SOM	227.18±25.04a	233.42±19.38a	231.51±20.33a	226.48±19.81a	215.92±27.79a	228.49±18.11a	225.11±26.58a	*P*=0.73
SBD	0.97±0.15a	0.91±0.12a	0.94±0.12a	0.97±0.15a	0.94±0.13a	0.92±0.14a	0.98±0.12a	*P*=0.07
Shannon	2.91±0.19a	2.67±0.13ab	2.79±0.19a	2.76±0.16ab	2.54±0.44b	2.77±0.27a	2.69±0.27a	*P*=0.64
Simpson	0.93±0.03a	0.89±0.02ab	0.91±0.02a	0.91±0.02a	0.86±0.09b	0.91±0.06a	0.9±0.04a	*P*=0.59
Pielou	0.89±0.05a	0.85±0.03a	0.87±0.03a	0.87±0.02a	0.84±0.09a	0.86±0.06a	0.86±0.05a	*P*=0.37
PD	1588.84±134.65a	1468.66±160.35a	1499.99±145.57a	1506.37±250.12a	1286.44±244.01b	1532.61±183.78a	1407.51±221.58b	***P*=0.03**
PE	72.17±45.20a	61.08±44.45a	63.07±36.68a	53.91±17.71a	43.33±15.49a	56.29±18.23a	61.12±45.48a	*P*=0.06
PSV	0.87±0.01b	0.87±0.01b	0.87±0.01b	0.87±0.01b	0.88±0.01a	0.87±0.01a	0.87±0.01a	*P*=0.79
Bacterial.shannon	8.96±0.22ab	8.87±0.19b	9.08±0.23a	8.87±0.13b	9.07±0.09a	8.98±0.24a	8.96±0.13a	*P*=0.14
Bacterial.PD	49.62±6.66ab	52.14±4.46a	53.94±6.32a	44.84±2.26b	49.42±3.63ab	50.27±6.42a	49.72±4.92a	*P*=0.14
Fungi.shannon	6.56±0.45a	6.38±0.66a	6.30±0.64a	6.14±0.63a	6.59±0.38a	6.15±0.48a	6.64±0.55b	*P*=0.35
Fungi.PD	50.36±5.72a	49.00±8.82a	48.05±7.13a	51.43±11.24a	52.09±9.33a	47.34±8.69a	53.03±7.27b	*P*=0.16
AGB	536.76±73.96a	550.57±101.13a	560.99±68.89a	505.62±98.22a	507.03±99.59a	529.32±75.42a	535.08±101.75a	*P*=0.28
BGB	834.48±319.59b	1229.95±337.54a	1170.11±394.93ab	881.85±254.04b	872.17±186.66b	993.13±220.02a	1002.29±431.79a	*P*=0.58
AGB/Total.biomass	0.40±0.08a	0.32±0.05b	0.35±0.05ab	0.35±0.07ab	0.36±0.05ab	0.35±0.05a	0.36±0.08a	*P*=0.98
BGB1	736.21±320.92b	1111.83±342.67a	1044.09±339.87ab	778.99±237.13b	773.89±204.27b	884.88±211.02a	893.13±411.26a	*P*=0.51
BGB2	65.78±21.53a	80.13±33.28a	91.19±59.68a	57.45±19.25a	68.17±23.59a	68.10±24.69a	76.99±43.73a	*P*=0.47
BGB3	32.57±14.93a	38.02±9.29a	34.99±8.82a	45.69±30.21a	29.89±6.57a	40.13±21.32a	32.33±8.88a	*P*=0.91
Total.biomass	1371.24±346.66b	1780.53±400.97a	1731.10±448.58ab	1387.46±244.95b	1379.20±253.34b	1522.44±236.56a	1537.37±492.69a	*P*=0.46
Com.stability	0.98±0.11b	1.33±0.47a	1.03±0.14b	1.06±0.12b	1.02±0.24b	1.02±0.23a	1.15±0.30a	*P*=0.12

Values are mean±SD (n = 5). Different letters in the same column indicate a significant difference at $P < 0.05$ among the treatments. W×G indicates the interaction of warming and grazing, and the number in W×G indicates the significant p-value.

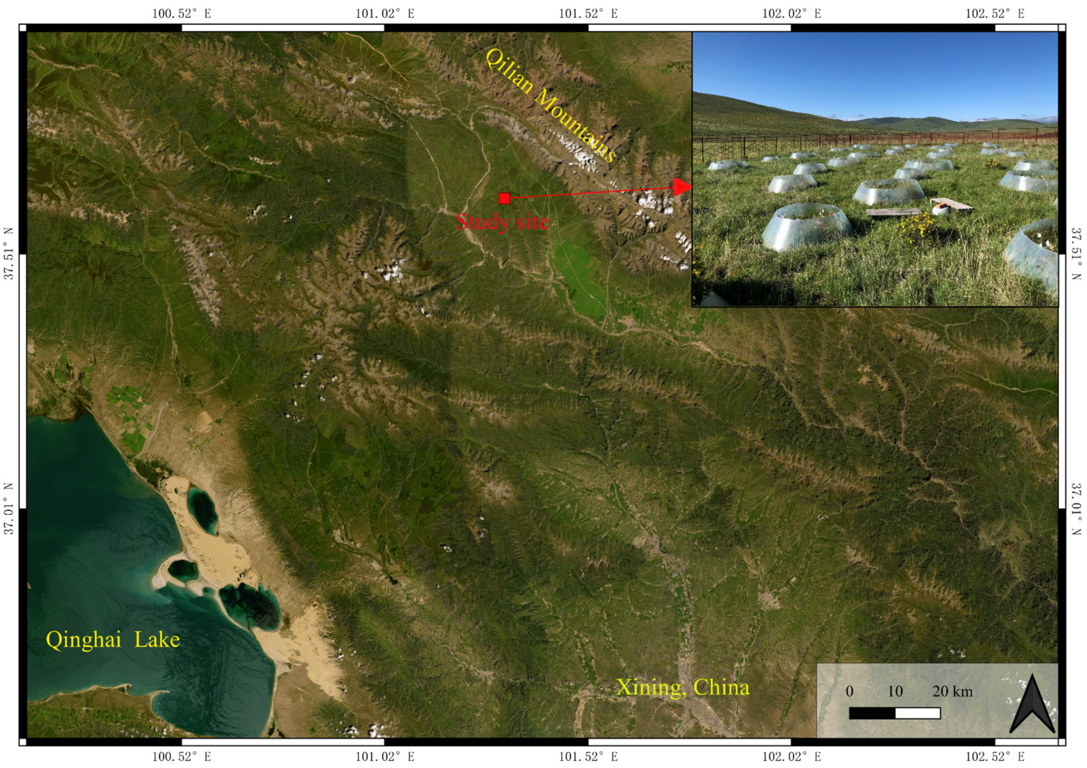

Figure S1 Location of the study site (Haibei Alpine Meadow Ecosystem Research Station) on the Qinghai-Tibet Plateau, China.

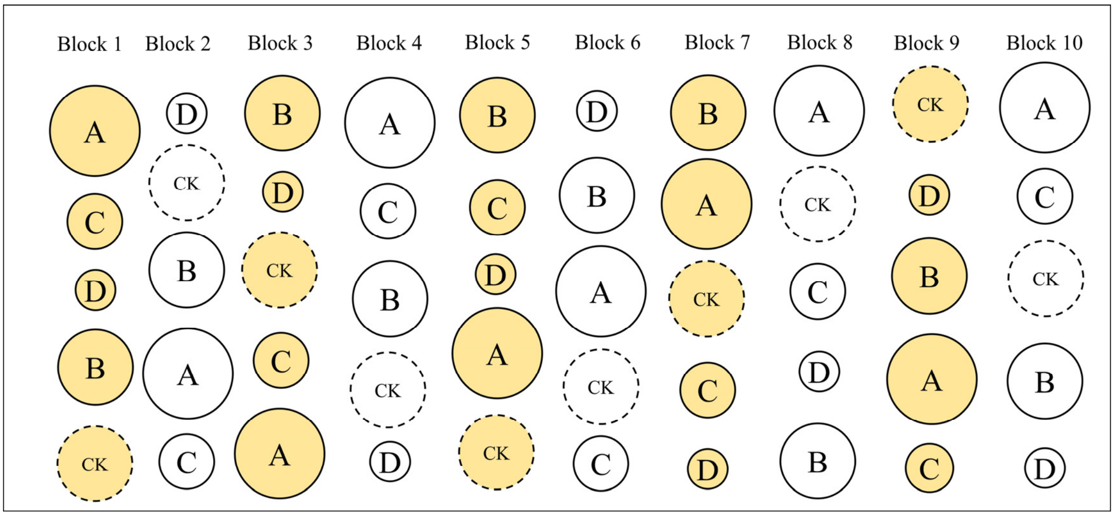

Figure S2 Schematic diagram of the experimental treatment design. This field experimental system of warming and grazing at the Haibei experimental Station of Northwest Institute of Plateau Biology, Chinese Academy of Sciences. Fifty plots composed of five temperature treatments (ambient temperature and four warming treatments) with ten replicates were distributed in 10 rows by 5 columns with a random block design. Each plot was separated from the others by a 2-m buffer strip. Half blocks with yellow mark were treated as stimulated grazing by clipping 60% standing litters before the growing season every year, and the remaining half were non clipping. Four levels of warming treatments were achieved by installing four types of conical open top

chambers (OTCs) constructed of 1.0 mm thick fiberglass.

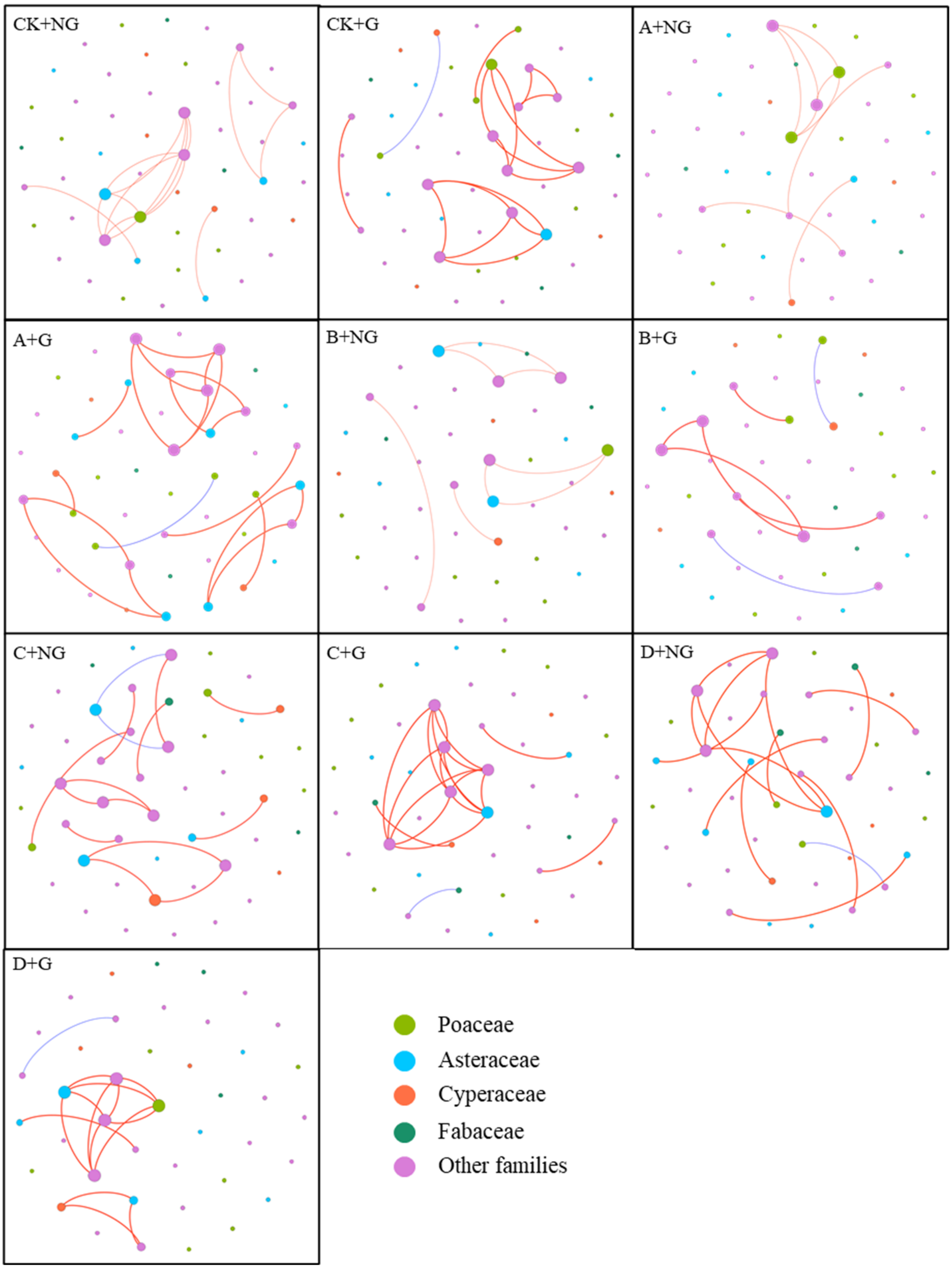

Figure S3 Network co-occurrence analysis of plant communities. The dots indicate species in the plant community, different colored dots indicate different plant functional groups. Lines indicate interrelationships between species, with red lines indicating positive correlations and blue lines indicating negative correlations.

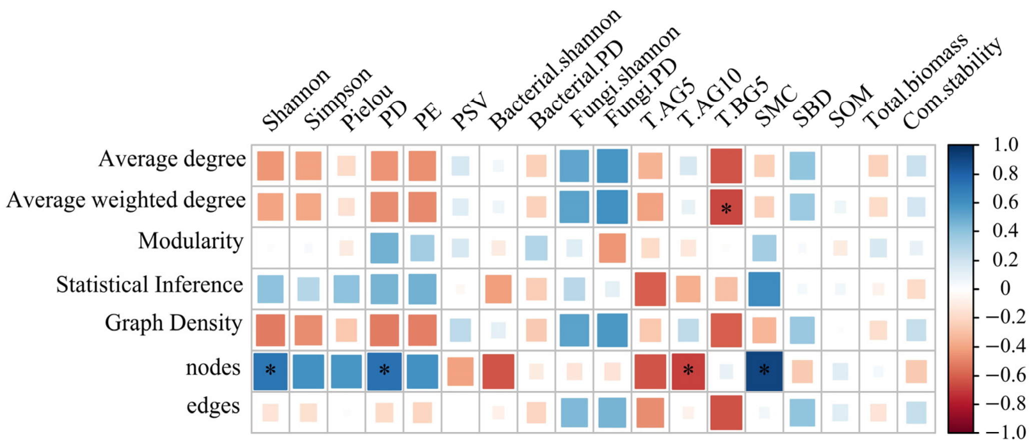

Figure S4 Correlation of co-occurrence network analysis results with biodiversity and ecosystem function
